# Whole Genome Sequencing and Evolutionary Analysis of Human Papillomavirus Type 16 in Central China

**DOI:** 10.1371/journal.pone.0036577

**Published:** 2012-05-04

**Authors:** Min Sun, Lei Gao, Ying Liu, Yiqiang Zhao, Xueqian Wang, Yaqi Pan, Tao Ning, Hong Cai, Haijun Yang, Weiwei Zhai, Yang Ke

**Affiliations:** 1 Key Laboratory of Carcinogenesis and Translational Research (Ministry of Education), Peking University Cancer Hospital & Institute, Beijing, China; 2 Center for Computational Biology and Laboratory of Disease Genomics and Individualized Medicine, Beijing Institute of Genomics, Chinese Academy of Sciences, Beijing, China; 3 Anyang Cancer Hospital, Anyang, Henan, China; National Institute of Health-National Cancer Institute, United States of America

## Abstract

Human papillomavirus type 16 plays a critical role in the neoplastic transformation of cervical cancers. Molecular variants of HPV16 existing in different ethnic groups have shown substantial phenotypic differences in pathogenicity, immunogenicity and tumorigenicity. In this study, we sequenced the entire HPV16 genome of 76 isolates originated from Anyang, central China. Phylogenetic analysis of these sequences identified two major variants of HPV16 in the Anyang area, namely the European prototype (E(p)) and the European Asian type (E(As)). These two variants show a high degree of divergence between groups, and the E(p) comprised higher genetic diversity than the E(As). Analysis with two measurements of genetic diversity indicated that viral population size was relatively stable in this area in the past. Codon based likelihood models revealed strong statistical support for adaptive evolution acting on the E6 gene. Bayesian analysis identified several important amino acid positions that may be driving adaptive selection in the HPV 16 population, including R10G, D25E, L83V, and E113D in the E6 gene. We hypothesize that the positive selection at these codons might be a contributing factor responsible for the phenotypic differences in carcinogenesis and immunogenicity among cervical cancers in China based on the potential roles of these molecular variants reported in other studies.

## Introduction

Human papillomaviruses (HPVs) are common and are clinically important pathogens [1]. Infection with high risk types of HPV is a necessary factor for the development of precancerous lesions and cervical cancer [2], [3], [4]. Of those that can infect human beings, over 120 different types have been isolated, and among these around 20 types are classified as high-risk HPV types (HR-HPV) based on their established association with cancer [1], [5], [6]. Among these high risk HPV types, HPV16 has been found to be the most prevalent and shows the strongest association with invasive cervical cancer [7], [8].

It is now generally accepted that HPV has co-existed with its human host over a very long period of time and has evolved into multiple evolutionary lineages [9], [10]. Intratypic variants of HPV16 have been identified from different geographic locations and are classified according to their host ethnic groups as European (including prototypes and Asian types), Asian American, African and North American [11], [12]. Through epidemiological and in-vitro experimental studies, natural variants of HPV16 have shown substantial differences in pathogenicity, immunogenicity and tumorigenicity. These variants may reflect the evolution of the viral population as it has adapted to local human ethnic groups [13]. By studying molecular evolution of the viral genomes, patterns of this evolutionary history can be identified and important molecular variants responsible for viral pathogenicity and carcinogenesis may be characterized [14].

There has been a paucity of HPV 16 population studies in China. Most previous studies have focused on studying the two major viral oncogenes E6 and E7 [15], [16], [17], [18], [19], [20], [21], [22], [23], [24]. The major goal of these studies has been to explore existing variants in the viral population. Although cataloging extant mutations is a necessary step in understanding HPV16 evolution, prioritizing the functional importance of these identified changes by examining their evolutionary pattern is potentially much more informative. In this work, we want to expand upon previous studies by characterizing the genome wide pattern of genetic diversity, and more importantly we want to pinpoint major genes/variants that are driving the adaptation of the virus to the human populations in central China. These evolutionarily important mutants may be used for further epidemiological and experimental studies where the functional consequences associated with these variants may be investigated and vaccines targeting these sites can be developed.

The nonsynonymous to synonymous rate ratio *d_N_*/*d_S_* in protein coding regions has provided an important means for studying molecular evolution of genes, and the use of this method has gained increasing popularity in recent years [25]. The basic rational of this method is that synonymous mutations do not change the underlying protein coding sequences and are not affected by natural selection. The synonymous substitution rate *d_S_* provides a natural measurement for the rate of evolution under neutral processes [26]. Since nonsynonymous mutations alter the underlying protein sequences and can be affected by natural selection, the relative magnitude of the nonsynonymous substitution rate *d_N_* to the synonymous rate *d_S_* provides a good means for studying natural selection [27]. Specifically, *d_N_*/*d_S_* >1 represents positive selection, *d_N_*/*d_S_* = 1 indicates neutral evolution, and *d_N_*/*d_S_* <1 implies there is purifying selection (or negative selection). Thus, the nonsynonymous to synonymous rate ratio *d_N_*/*d_S_* provides a proxy for studying natural selection acting on coding genes, and many statistical methods have been developed to look for genes which are under the influence of natural selection, particularly Darwinian positive selection [28], [29].

Recent development of codon based substitution models has provided a natural extension of previous methods by allowing different codons to have different *d_N_*/*d_S_* values [30], [31]. Statistical methods such as the likelihood ratio test can be employed to determine whether patterns of molecular evolution at a certain gene can be explained with models without invoking positive selection [32], [33]. Upon rejecting the null hypothesis in favor of the alternative model where positive selection is explicitly allowed, special codon positions under adaptive evolution can be identified using a Bayesian based approach [33], [34]. These methods have been widely applied to many datasets, including some multiple whole genome sequences [35].

In this work, we took a whole genome approach and sequenced 76 HPV16 isolates from Henan Province, China (located in central China, see Figure S1). We wanted to determine whether any of the genes in the HPV16 genome is driven by positive selection. In addition, we sought to identify those codon positions and associated amino acid changes responsible for the adaptive evolution in this viral population.

## Materials and Methods

### Sample collection

HPV viruses often have low concentrations in normal tissues and are difficult to amplify. In this study, ninety four paraffin-embedded blocks of cervical cancer samples were collected to extract the viral genomes from the human population. Of these ninety four samples, seventy six tested HPV16 positive and were used for subsequent sequence analysis. These tissue specimens were collected from women with cervical cancers during their primary treatment between 2005 and 2007 at Anyang Cancer Hospital, Henan province, China (Figure S1). All the patients received no chemotherapy before the surgery. The tumor samples in this study were a small proportion of the patient samples from this hospital where surgery (i.e. removing uterus) was chosen as an effective treatment. Later stage cancer patients will directly go to radiation therapy without surgery. The clinical stage and associated age information for these patients were presented in the supplementary information (Table S1). Official approval from the Institutional Review Board of Peking University School of Oncology, and an informed consent was signed by each patient before sample collection.

### DNA preparation

5 µm paraffin sections of formalin-fixed tissue were de-paraffinized in xylene, and washed with 100%, 95%, and 75% ethanol. The tissue was pelleted, air dried and digested with proteinase K (200 mg/ml) at 55°C overnight. 200 ul of this material was isolated using an H.Q. & Q. Tissue DNA Kit (U-GENE BIOTECHNOLOGY CO., LTD, Anhui, China). DNA was re-suspended in a final volume of 100ul 10 mM Tris. The DNA concentration was determined by use of a Nano-Drop (NanoDrop Technologies, Wilmington, Delaware USA). A full description of sample processing and DNA extraction were presented in great detail in supplementary materials (Text S1).

### Quality Control

Our experimental work followed strict quality control to avoid possible contamination from lab environments. As presented in great detail in a previous study [36], DNA extraction, PCR reaction and DNA electrophoresis were done in separate rooms and specimens moved only in one direction. Laboratory personnel were instructed to wear gloves when handling the samples and the experimental area were regularly cleaned before beginning work. In addition, a routine procedure of inspecting the experimental area surfaces (cotton bud was first applied to various of surfaces, e.g. lab benches, subsequently they were soaked into deionized water overnight. HPV detection was applied to the supernatant. Experiments were allowed only when negative results were observed). Additionally, we also used a mouse liver tissue as an internal control together with the cancer samples. Experiments were preceded only negative results were observed from these internal controls (Text S1) and also our previous study [36].

### HPV DNA Detection and HPV16 DNA identification

A modified set of primers, SPF1/GP6+, which amplify an L1 fragment of approximately 184 bp were used. The polymerase chain reaction was carried out as follows. Qiagen Hot Start Taq DNA polymerase mixture was used with 4 mM MgCl_2_, and 10 pmol of each primer. The activation of the enzyme was carried out at 95°C for 15 minutes, followed by 40 amplification cycles at 95°C for 40 seconds, 49°C for 50 seconds, 72°C for 30 seconds, and a final extension at 72°C for 5 minutes.

The presence of HPV16 DNA in the L1 positive samples was evaluated by type-specific PCR which amplified a 335bp (nt231 to 565) fragment of HPV16 E6. PCR was performed at 95°C for 15 minutes, followed by 40 amplification cycles at 95°C for 40 seconds, 57°C for 40 seconds, 72°C for 40 seconds, and a final extension at 72°C for 5 minutes. The experimental conditions and amplification regions are presented in the supplementary materials (Text S1 and Table S2, S3 and S6).

### PCR and Sanger sequencing

PCR primers were designed to cover the HPV genome. Platinum Taq DNA polymerase High Fidelity (Invitrogen Co., Carlsbad, CA, USA) was used for PCR experiments (Table S5 and S6). PCR products were purified using a PCR clean-up gel extraction column (MACHEREY-NAGEL GmbH & Co, Düren, Germany) according to the manufacturer's instructions and were directly sequenced using a capillary sequencer (ABI Prism 3100).

For this study, in addition to the quality control listed above, five specimens were chosen to repeat the experimental procedures (including sample processing, Text S1). A different primer sets were used to amplify the HPV genome (Table S4). The PCR products were purified and ligated into the pEASY-T1 vector (Transgen Biotech Co. LTD, Beijing, China) and 3-5 colonies per ligation were subsequently sequenced with the using the same Sanger method. The PCR primers and reaction conditions are presented in the supplementary materials (Text S1, Table S4 and S6).

### Sequence alignment and phylogenetic reconstruction

For each sample, sequence segments across the HPV genome were concatenated into a single genome. Alignment software MUSCLE [37] was used to align these genomes against each other with default parameters. The corresponding annotation information was extracted by comparing the sequence alignment to the HPV 16 reference genome [38], [39] (see data availability). Phylogenetic relationships were built using PhyML with the General Time Reversible (GTR) model and gamma distributed rate variation among sites [40]. Local phylogeny for each gene was also constructed with the same procedures. In order to access the confidence in the phylogenetic relationship, non-parametric bootstrap analysis was also carried out using PhyML and summarized with the SUMTREES package [41].

### Population analysis

We carried out a sliding window analysis on the genetic diversity along the HPV genome using custom written python scripts (available upon request). The genetic diversity was estimated based on both the Watterson and the Tajima methods [42], [43]. For a focal window, Watterson's estimator of genetic diversity uses information from number of polymorphic sites. Specifically, Watterson's estimator of genetic diversity is 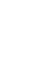, where S is the number of polymorphic (or segregating) sites, and n is the sample size [43]. Likewise, Tajima's estimator of genetic diversity is the average pairwise differences between two sequences taken at random from the sample [42]. Both estimators capture the genetic diversity within a sample gathered from a population with slightly different weight on sites at different frequencies. For a standard equilibrium population, these two estimators should obtain similar values. Differences between estimated values implies either non-equilibrium populations (e.g. past population growth or bottleneck) or occurrence of natural [selection 42].

### PAML analysis

CODEML from the PAML package was used to look for the signal of positive Darwinian selection across the HPV genome. In particular, we used the M1a/M2a model and the M7/M8 model to construct the likelihood ratio test for detecting positive selection [44]. In brief, in the M1a model (null model), there are two categories of sites with different omega (the nonsynonymous rate to synonymous rate ratio or *d_N_*/*d_S_*) values. One category has an omega value between zero and one, representing the set of codons evolving under purifying selection. The second category has an omega value of 1.0, corresponding to those sites under neutral evolution. In the alternative model (M2a), an extra category of sites with omega values greater than one is added. If the alternative model provides significant improvement in the likelihood in supporting the alternative model (Likelihood Ratio Test or LRT), the gene under study is said to have sufficient statistical support for existing of positive selection [32], [33].

In the M7 model, omega values are constructed to follow a beta distribution between zero and one. In the M8 model, one extra category of omega with value greater than one is added to the model to allow for positive selection. The likelihood ratio test can also be constructed with the M7/M8 models to test for positive selection. In general, nested test between M1a/M2a is more robust/less powerful than M7/M8 comparisons, even though most of the time, they give very similar results [44].

In the likelihood ratio test, twice the log likelihood difference between the two models is compared with the chi-square distribution wherein the degree of freedom is equal to the differences in the number of free parameters between the two models. In both the M1a/M2a and M7/M8 comparisons, two degree of freedoms should be used (both the omega parameter and proportion of sites for the extra category). Upon rejecting the null hypothesis in favor of the alternative model with positive selection, Bayesian Emprical Bayes (BEB) procedures can be used to identify the set of sites under positive selection [34].

## Results

### HPV infection and PCR amplification

With the carefully designed PCR primers, we were able to detect HPV in 87 (92.5%) of the 94 cancer samples. Of these 87 samples, 80 cases were positive for HPV16. In addition to type 16, other HPV types were also detectable at low frequencies (Text S1). Of the 80 cases that were HPV16 positive, we were able to extract HPV 16 DNA sequences from 76 samples. HPV concentrations in four other cases appeared to be too low for efficient PCR reactions.

Following the PCR reactions, Sanger sequencing was conducted for all the PCR products. In order to check the quality of the experiments, five specimens were chosen to repeat the lab procedures with clone sequencing (instead of direct PCR/Sanger sequencing). Three to five colonies per ligation were cloned and subsequently sequenced from both ends using traditional Sanger methods. Based on regions which overlapped by more than one PCR region and multiple colonies per ligation, consistent results were found for each sample/region and match with our results from direct PCR/Sanger sequencing. This suggested that type 16 HPV was the predominant type in these cancer patients and diversity within hosts was not substantial. Except for three regions (475 bp and 309 bp in E1 in all 76 samples and 928 bp in E2 in 31 samples), all other parts of the HPV genome were successfully amplified and sequenced. The undetectable dosages in parts of the E1/E2 regions could either due to PCR failures (see discussions) and might also reflect the viral genome integration typically found in cancer samples [45], [46] and the low concentration of the episomal form of the viral particles in the samples. The technical aspects of amplification and sequencing are presented in detail in the supplementary materials (Text S1).

### Phylogentic relationship

**Figure 1:**
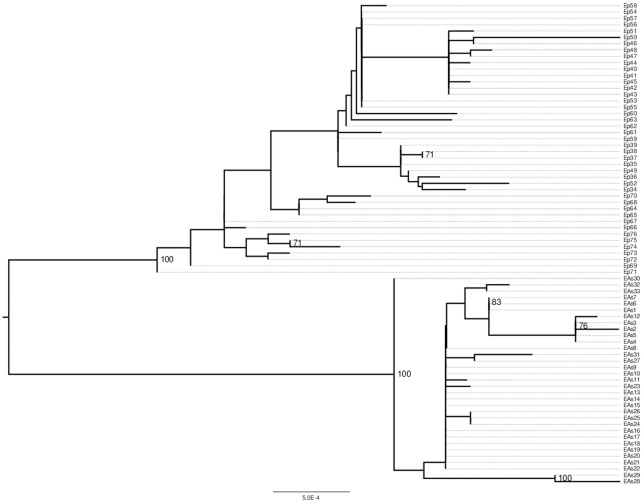
Maximum Likelihood phylogenetic tree for 76 HPV16 samples from Anyang. This tree is constructed using whole genome sequences and bootstrap scores larger than 70% are displayed. The tree itself is shown in bold, the sample IDs are linked through dashed lines. EPs are the European Prototypes and EAs are the European Asian types.

After retrieving these sequences, the computer software PhyML was used to reconstruct the phylogenetic relationships among the 76 samples under a General Time Reversible (GTR) model of nucleotide substitution and gamma distributed rate variation among sites. The resolved maximum likelihood tree is shown in Figure 1. This figure illustrates the set of sequences which are grouped into two major clades including European prototype (E(p)) and the Asian (E(As)) type, with the strong statistical support of a bootstrap value of 100% among the 500 replicates. The observed frequency of the Asian type is 43.4% (33 out of 76) which is within the range of values observed in previous studies within China [18], [19], [21], [23], [24].

In addition most of the differences within each variant (E(p) or E(As)) are quite small as compared with the divergence between variants (Figure 1). This pattern indicates a population history wherein two viral subpopulations shared some evolutionary history in the distant past and subsequently diverged from each other [12]. This may reflect earlier divergence in HPV 16 viral populations as they were adapting to different human ethnic groups.

### Genetic diversity

**Figure 2:**
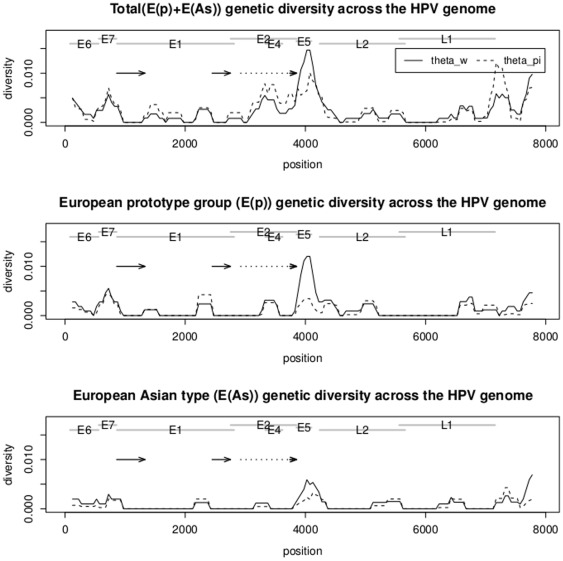
Sliding window plot of genetic diversity across the HPV genome. Window size is set as 250 bp and step size = 50 bp. The solid arrows indicate the two regions in the E1 gene that failed in the PCR reactions. The dashed arrow indicates the region in E2 gene that failed in some of the specimens. Theta_w is the Watternson's estimate of genetic diversity which is based on the number of polymorphic sites [43], and theta_piis the Tajima's estimate of genetic diversity relying on average pairwise differences [42].

As compared with many RNA virus such as HIV or influenza virus, the HPV 16 population shows relatively low diversity across the viral genome (summarized in Table 1 and also Figure S2). Mean pairwise differences between any two sequences across the HPV genome are often less than 0.01 (see Figure 2). As compared with many RNA viruses, this level of diversity is quite small [47]. In addition, the genome-wide variation in genetic diversity correlates well with previous observations based on datasets gathered from other geographic locations [12], [14]. The highest genetic diversity was found to be around the E4/E5/NCR regions and in the URR/E6/E7 regions. After splitting into subgroups, European Prototype group shows slightly higher genetic diversity than European Asian types (Figure 2).

**Table 1:** Summary of the polymorphisms in HPV genes

Gene	Polymorphic sites	Nonsyn		Syn		Gene size(bp)	Theta_W^c^	Theta_Pairwise^d^
		Ts^a^	Tv^b^	Ts	Tv			
E6	7	2	4	1	0	456	0.0030	0.0019
E7	7	2	1	4	0	297	0.0049	0.0060
E1	6	3	0	3	0	1950	0.0010	0.0012
E2	12	4	6	2	0	1098	0.0022	0.0034
E4	5	0	0	3	2	288^e^	0.0036	0.0024
E5	15	5	4	3	3	252	0.0123	0.0061
L2	9	2	3	4	0	1422	0.0013	0.0007
L1	8	1	1	4	2	1596^f^	0.0009	0.0005

a,b : Transition and transversion mutations.

c : Watternson's estimate of genetic diversity based on the number of polymorphic sites [43].

d : Tajima's estimate of genetic diversity based on average pairwise differences [42].

e,f: We found an alternative annotation in the literature [74]. The corresponding numbers are 279 and 1518 respectively.

A full display of all variants was given in the supplementary Figure S2.

Comparing the two measurements of genetic diversity, Watterson's estimate based on the number of segregating sites is close to Tajima's estimate which is based on pairwise differences. Similar measurements of these two suggest a genealogical pattern that is typically found in an equilibrium population. This implies that the effective viral population size in the recent past has been quite stable [42], [48]. This is consistent with the observation in human population that despite the world wide human census population size has increased quite rapidly in recent history, the effective human population size is relatively stable.

### Signals of positive selection

There are two major sources of adaptive forces which act on viral genes over their life history. One source of driving force is host immune surveillance. Mutations that lead to escape from immune recognition by the host immune system (class I and II molecules) often provide viruses with increased fitness (higher surviving rate). The second source is viral gene function. Mutations that result in viruses with increased functional ability (e.g. increased enzymatic activity or binding affinities on downstream targets) are often adaptive. These two forces represent the “attack and defense” aspects of viral life history and embody two sides of the same coin. In reality, these two sources often overlap due to the pleiotropic effects of viral genes.

The nonsynonymous to synonymous rate ratio (often denoted as omega, *d_N_*/*d_S_* or *K_a_*/*K_s_*) measures the relative ratio of the nonsynonymous to synonymous evolutionary rate and is a good indicator of selection. In particular, positive selection favoring nonsynonymous change will lead to an elevated nonsynonymous substitution rate relative to the synonymous rate. other words, positive selection will result in *d_N_*/*d_S_* values greater than one. On the other hand, negative selection (or purifying selection) acting to preserve certain amino acid positions will give *d_N_*/*d_S_* values of less than one. If nonsynonymous substitutions are relatively neutral, the *d_N_*/*d_S_* value will be close to one. The CODEML software package was used to apply a wide range of statistical models to test for positive selection. The results for the likelihood ratio test using two sets of models for evaluation of all HPV genes are listed in Table 2, and most of these genes showed only a very weak signal or no signal for positive selection (Table 2). The only gene for which evidence of positive selection reached statistical significance is the oncogenic E6 gene. Gene E1 showed marginal significance at the level of 0.1 (p value = 0.09). A few interesting codons were identified in this analysis in a few other genes, even though these genes did not reach statistical significance due to the small number of changes (Table 1). These results will be discussed in detail with regard to their functional significance in the following section.

**Table 2:** Likelihood ratio test for the eight genes across the HPV genome

	lnL(Null model)	lnL(Alter model)	-2lnL (pvalue)	Positive selected sites(BEB posterior pvalue)
**E1**	−1529.57 (M1a)	1528.10(M2a)	2.95 (0.09)	491(0.772)
	−1529.57 (M7)	−1528.10(M8)	2.95 (0.09)	186 (0.529),326(0.529), 491(0.855)
**E2**	−1528.99 (M1a)	−1528.92 (M2a)	0.14 (0.71)	25 (0.502),165(0.505), 208(0.504),219(0.510)
	−1528.99 (M7)	−1528.92 (M8)	0.14 (0.71)	25(0.716),135(0.713), 165(0.718), 173(0.709), 208(0.717), 210(0.714),219(0.722), 232(0.708),310(0.713), 344(0.713)
**E4**	−406.13 (M1a)	−406.13 (M2a)	0.000(1.00)	-
	−406.13 (M7)	−406.13 (M8)	0.000(1.00)	-
**E5**	−419.67 (M1a)	−419.67 (M2a)	0.000(1.00)	44 (0.503)
	−419.67 (M7)	−419.67 (M8)	0.000(1.00)	44 (0.655)
**E6**	−681.92 (M1a)	−677.38 (M2a)	9.08**(0.003)	10(0.957*),25(0.995**), 83(0.995**),113(0.957*)
	−681.93 (M7)	−677.38 (M8)	9.08**(0.003)	10(0.985*), 25(0.999**), 83(0.999**),113(0.985*)
**E7**	−443.79 (M1a)	−442.58 (M2a)	2.42 (0.12)	29(0.723)
	−443.79 (M7)	−442.58 (M8)	2.43 (0.12)	29(0.832)
**L1**	−2162.25 (M1a)	−2162.25 (M2a)	0.000(1.00)	-
	−2162.26 (M7)	−2162.26 (M8)	0.000(1.00)	-
**L2**	−1860.35 (M1a)	−1860.35 (M2a)	0.000(1.00)	-
	−1860.35 (M7)	−1860.35 (M8)	0.000(1.00)	-

All sites with posterior probability (BEB) greater than 0.5 were listed in this table, * significance at 95% level, ** significance at 99% level

## Discussion

### Functional significance of the genes and codons

Several of the amino acid positions identified in the E6 gene are of significant interest. During the host immune response, viral peptides are often presented to the host immune system (e.g. cytotoxic T-Lymphocyte CTL) through antigen presenting functions mediated by Major Histocompatibility (MHC) molecules. Previous studies have found that mutations in the amino acid position L83V may play an important role in cancer progression. For example, the L83V polymorphism located within the epitope which binds to MHC molecules was found to be associated with cervical tumor development [49], [50]. This variant can also promote neoplastic transformation depending on the host genotypes at MHC class I and II loci [50], [51], [52].

On the other hand, the E6 gene is also an important oncogene which binds to the E6-AP (host E6 associated protein). E6-AP's ubiquitin ligase activity functions to ubiquitinate p53 and subsequently leads to P53 proteosomal degradation. The E6 oncogene has been shown to promote transformation of immortalized human epithelial cells. A previous study found that L83V appears to enhance MAPK signaling and L83V is involved in oncogenic Ras-mediated transformation [53], efficient degradation of Bax and binding to E6BP and decreased binding to human discs large protein (hDlg) [54], [55]. These functional changes are thought to give HPV higher carcinogenic potential. In addition, this L83V polymorphism also appears to interact with natural human variations in the P53 genes (in particular codon 72 polymorphism) to confer differences in cervical cancer risk [56].

The other positively selected site of interest is amino position D25E. Polymorphism at this site has been found to be relatively rare in western countries, but occurs at higher frequency in Asian populations [15], [23], [57], [58]. Polymorphisms at this site are also found to interact with human HLA polymorphisms to contribute cervical carcinogenesis [57], [58].

In addition to amino acid positions 83 and 25 discussed above, several other sites have also been studied. For example, polymorphisms at codon position 10 seem to interact with the HLA-B7 peptide binding epitope and influence immune recognition through CTL [52]. Position E113D mutation was also implied in invasive cervical carcinoma [11], [23].

It is noteworthy that the other oncogene E7 showed a signal of positive selection which was considerably reduced as compared with E6 protein. This observed higher conservation of the E7 protein appears to be quite general across world wide populations and at many different evolutionary scales [12], [14], [59], [60], [61], [62] (see discussions). It is of interest that the only candidate site which was positively selected N29S is located within the domains which are important for the transforming activity of this protein and these domains are know to be involved in binding retinoblastoma suppressor protein (pRB) [63]. In addition, this position is also present within the protein's immunoreactive regions and may also be involved in both immune recognition and oncogenicity of the virus [64].

Other than these two important oncogenes, a few other loci are also worth discussion. In the likelihood ratio test, the E1 gene showed marginal significance at a level of 0.1. The E1 protein plays an important role in viral replication-associated activities such as origin-specific binding and helicase activities, and it forms a complex with the E2 transactivator. It is interesting to note that the positively selected site 491 identified in E1 protein is located in the E2 binding domain and can bind to DNA polymerase alpha-Primase p68 Subunit [65].

The likelihood ratio tests on the E2 gene show a very weak signal of positive selection. However, both sets of models identified a few potential candidate sites to be under positive selection. During early viral infection, the E1 and E2 proteins bind jointly to the DNA at the origin of replication. The papillomavirus E2 protein is required for viral replication and regulates both viral transcription and replication, and therefore plays a central role in the viral life cycle. In addition, E2 is also important for repressing oncoprotein transcription.

The E2 protein can be partitioned into three major functional domains. The transactivation domain which is engaged in E1 interaction and TFIIB interaction, the linker domain and the DNA binding domain [66]. The DNA binding domain is responsible for E2 dimmerization, E1 interaction and DNA recognition. In our analysis, many codon positions including 25, 135, 165, 173, 208, 210, 219, 310, 344 in the transactivation domain and the DNA binding domain all showed some weak evidence of positive selection even though they haven't reach statistical significance of 0.95 due to limited number of changes. Since these sites are all involved in the replication process, we could imagine selection in these positions (including some of the positions in E1) may be involved in the fine tuning the efficiency of DNA replication. For example, E2 T310K has been linked to high grade histology in cervical carcinoma [67]. Definitively answering the questions about positive selection in E2 protein is still challenging with our current study due to limited power in our data (i.e. small numbers of changes). Further studies with larger sample sizes might be able to look into these questions, especially the role of positive selection, in greater depth.

The other common observed phenomenon is that E2 breakage and HPV integration are highly correlated with neoplastic progression [46]. Integration typically happens late in the infection cycle and has been shown to be associated with tumor development [45]. Whether these mutations are functionally linked to the integration process is currently unknown and warrants further study.

### Frequency comparisons with other populations

When an advantageous mutation arises in a single virus, it will quickly increase in frequency and spread through individual local populations owing to its favorable fitness (i.e. selective sweeps in population genetic terms) [68]. This will lead to higher genetic differentiation between population groups at these loci. In Table 3, we compiled frequencies extracted from several previous studies for the positively selected positions in the E6/E7 genes. It is clear that these frequencies vary widely among different human populations. The wide-ranging differences in allele frequencies are consistent with our expectations based on population genetic theory, even though genetic drift could also contribute to the observed differences. Whether this divergence is associated with CaCx pathogenesis in these populations needs further investigation.

**Table 3:** E6/E7 positively selected sites and their associated frequencies curated from previous studies

Population	Type	Sample size	E6				E7	Reference
			R10G	D25E	L83V	E113D	N29S	
Anyang^a^	Cancer	76	1.32	42.10	5.26	1.32	43.42	This study
Hubei^a^	Cancer	72	0	62	6	9	-	15]
Wenzhou^a^	Cancer	55	0	67.2	9.1	0	-	19]
Sichuan^a^	Cancer	113	0	31.0	31.0	6.2	-	20]
Taiwan^a^	Cancer	17	0	88.2	0	64.7	88.2	17]
Jiangxi&Guangdong^a^	Cancer	55	0	67.3	3.64	9	70.2	23]
HongKong^a^	Cancer	255	0	50.6	7.1	4.3	58.0	16]
Beijing^a^	Cancer	31	0	41.94	19.35	6.45	45.16	24]
Korea	Cancer	27	7.4	85.2	3.7	7.4	-	75]
Japan	Cancer	43	0	44	33	26	-	58]
India	Cancer	60	0	0	55	0	0	76]
Thailand	Cancer	31	0	87	19.3	0	100	77]
Indonesia	Cancer	22	0	9.1	9.1	0	22.7	78]

a : Places in China, also see Figure S1.

-: not reported.

High risk HPV16 infection plays an essential role in the carcinogenesis of CaCx and other tumors. Intratypic HPV16 variants isolated from different geographic regions and ethnic groups have shown varied biological and pathological properties. Epidemiology studies have shown particularly increased risk for the development of cervical lesions associated with non-European variants of HPV16 [69], [70], [71]. In vitro experimental studies have demonstrated variability in the biological properties of HPV16 variants which may account at least in part for differences in viral pathogenicity, risk of carcinogenesis, and immunogenicity [72]. An evolutionary analysis of the variants of HPV can reveal the selective pressures on individual genes and codon positions and may therefore guide epidemiological and functional studies. A genome wide approach as presented in this study provides one of the first investigations of HPV16 evolution in Central China.

Several of observations from the current study have added to our previous knowledge of HPV evolution. First, the genome wide genetic diversity observed in the Anyang area is largely concordant with previous studies of the papillomavirus family. The higher degree of diversity observed around the E4/E5/NCR regions and URR/E6/E7 regions seem to be found consistently for the PV family in general [59], [61], as well as in human PVs [60] and within HPV16 [62]. Elevated genetic diversity might be due to a higher local mutation rate, but may also be the result of selection over the course of the viral life history for its function in genome replication or expression. Definitively separating and determining the relative influence of these two factors will require further studies. However, it is of significant interest to observe that this concordance in genetic diversity is conserved at multiple levels across million of years of evolution.

Secondly, most previous studies that have investigated HPV16 in cervical cancer tissues have shown that a majority have integrated genomes. Integrated genomes should result in amplification of some regions, but not in amplification of the entire HPV16 genome [45], [46]. Out of the 76 samples studied here, 31 specimens failed in PCR amplification of the E2 region. This proportion is likely an underestimate of the percentage of the integrated form within the cancer samples we sampled, because mixture of episomal and integrated form of HPV16 may also allow effective amplification the E2 region. However, considering PCR failure could also be a potential confounding factor even though we did repeated many times to reduce possible false negatives, definitive conclusions about the proportions of integrated forms remains challenging for our study. Nevertheless, since we are aiming at characterizing the within population diversity, and assume that there is no strong correlation between genome integration and HPV genotypes, our observations are unlikely to be biased by the physical status (integrated versus not integrated) of the viral genomes.

Lastly, the codon based likelihood models utilized in this study are based on measuring the relative magnitudes of nonsynonymous and synonymous substitution rates, which relies on having sufficient evolutionary changes over the history of the sample. The evolutionary divergence observed in this area is relatively small (Figure 1) and is likely to affect the sensitivity of the method. In other words, the results presented in Table 2, especially those regarding marginally significant sites/genes might be affected by statistical power. For example, using all major lineages of HPV16, which presumably included much higher levels of diversity than our work, one of previous studies also found strong statistical support for positive selection in both E5 and E6 genes [14]. Even though possible adaptive evolution happened during divergence between major HPV16 lineages (e.g. European vs non European types) is very likely, statistical power due to limited changes might also be partially contributing to the observed slight differences. Nevertheless, it is quite reassuring that many of the findings presented in this work coincide well with many previous studies [14].

It is worth pointing out that the samples we collected are still solely from the cancer tissues, which might lead to biases in representing the general landscape of the HPV variations in this region. However, considering the difficulties in sequencing the HPV genomes in the normal tissues, our study is still a worth-while step towards such an unbiased study. With the forthcoming high-throughput sequencing techniques, whole genome analysis of the viral population is becoming increasingly attainable. Especially promising in this regard is the potential for sequencing large genomic segments of several kilobases with single-molecules using real-time sequencing technologies [73]. The study presented in this article is one of the first steps in studying the HPV populations in China. Similar further research across many human populations may draw a much more complete picture of HPV16 evolution in human beings. These studies will guide further epidemiological and functional studies aimed at understanding HPV life history, pathogenicity and immunity.

### Data availability

The sequence data presented in this study will be available on our public ftp site at ftp://ftp.big.ac.cn.

HPV reference links http://www.stdgen.lanl.gov/papilloma/GenBank-files/Human-papilloma/HPV16R.gb

## Supporting Information

Figure S1 **A geographic map of South East Asia with Anyang and several other locations (** **Table 3** **, maintext) marked.**
(TIFF)

Figure S2 **Nucleotide variations across the HPV genome for our sample.**
(PDF)

Table S1 **HPV16 positive cervical patient information.**
(PDF)

Table S2 **PCR primer for testing HPV presence.**
(PDF)

Table S3 **PCR primers for testing HPV types.**
(PDF)

Table S4 **The PCR primers for the five samples that were checked with clone sequencing.**
(PDF)

Table S5 **The PCR primers for sequencing the HPV16 genomes.**
(PDF)

Table S6 **PCR reaction conditions.**
(PDF)

Text S1 **Supporting methods.**
(DOC)
