# Supplementary figures and images for: Whole Genome Sequencing and Evolutionary Analysis of Human Papillomavirus Type 16 in Central China
Source: PLoS One. 2012 May 4;7(5):e36577. doi: 10.1371/journal.pone.0036577 (PMC3344914; doi:10.1371/journal.pone.0036577)

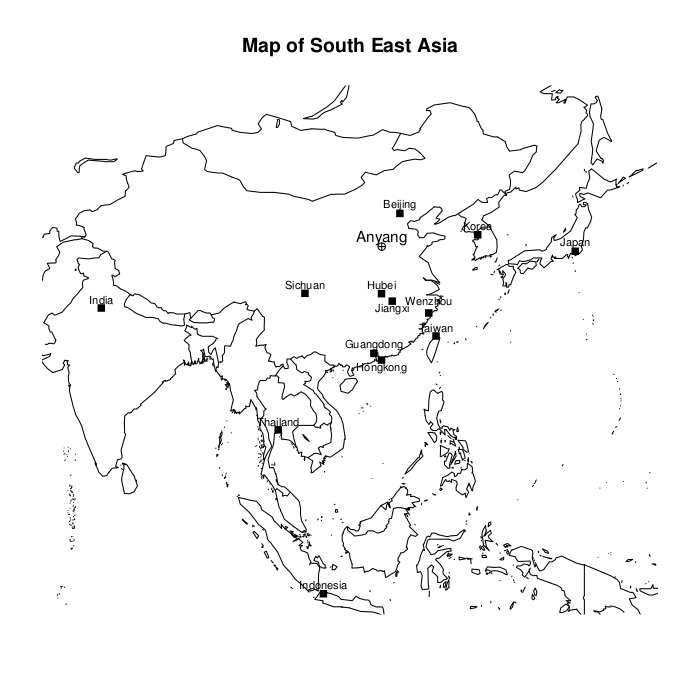

Supplement: Figure S1 — A geographic map of South East Asia with Anyang and several other locations ( Table 3 , maintext) marked. (TIFF) [file pone.0036577.s001.tiff]
