# Supplementary material for: Whole Genome Sequencing and Evolutionary Analysis of Human Papillomavirus Type 16 in Central China
Source: PLoS One. 2012 May 4;7(5):e36577. doi: 10.1371/journal.pone.0036577 (PMC3344914; doi:10.1371/journal.pone.0036577)
Supplement: Figure S2 — Nucleotide variations across the HPV genome for our sample. (PDF) [file pone.0036577.s002.pdf]

Figure S2. Nucleotide variations across the HPV genome for our sample

```
                      1112222333333333333333333444444444444444444555556666667777777777777777777777
          1113466677884582338122334456789999999000000001111123337002453566680112222344677777778888
       2993775444669442145342547781428843356778011447790117903595452610056826770788819700138990245
       4341680267600361524748994740944770823295639031727196123688106680587960571079796023401292625
Ep_ref CAGAGTTAAAGTCTTTGATGCGCGTCTCGTCCTAGTAAAGGCTTTAAAAATCTTATTTTGAAACAAATCGATTCAAGATAGGTATCGCGGT
1      --A---G----C---N---NN------T----C-----------------------N-NNC--------N---------------------
1      --A---G-C----------NN------T----C-------------------------C-C--------A---------------------
1      -TA---G-C------N---NN-NNNNNNNNNNN-----C-----------------N-NNC--------N---------------------
1      --A------------N---NN------T----C-----C------C----------N-NNC--------N---------------------
1      --A------------N---NN-NNNNNNNNNNN------------C----------N-NNC--------N---------------------
1      ------------T--N---NN------TA---C-----C-----------------N-NNC--------N---------------------
1      ----------A----N---NN------T----C-----C-----------------N-NNC-----T-TN---------------------
1      ------------T--N---NN-NNNNNNNNNNN-----------------------N-NNC----NN--N---------------------
1      ----------A----C---NN------T----C-----------------------N--AC------GTA---------------------
1      ---------------N---NN-NNNNNNNNNNN-----------------------N-NNC----NN--N---------------------
1      ---------------N---NN------T----C-----------------------N-NNC--------N--------C------------
2      ----------A----N---NN-NNNNNNNNNNN-----------------------N-NNC----NN--N---------------------
1      ---------G----CN---NN-NNNNNNNNNNN-----------------------N-NNC----NN--N----------AAG--------
1      ---------------N---NN-NNNNNNNNNNN-----------------------N-NNC----NN--N------------G--------
1      ----A-G--------N---NN------T----C-----------------------N-NNC-----C--N------------G--------
1      ---------------N---NN-NNNNNNNNNNN----G--T---A-----A-----N-NNC-----C--N------------G--------
1      ---------------N---NN------T----C-----------------------N-NNC-----C--N------------G--------
1      ---------------N---NN-NNNNNNNNNNN-----------------------N-NNC-----C--N------------G---C----
1      ---------------N---NN------A----C-----------------------N-NNC-----C--N------------G--------
1      ---------------C-----------A----C---------------------------C-----C---------------G--------
3      ---------------N---NN-NNNNNNNNNNN-----------------------N-NNC-----C--N------------G--------
1      ----------A----------------A----C--C-------C------------CC-AC-----C---------------G--------
1      ----A------C---N---NN-NNNNNNNNNNN-----------------------N-NNC-----C--N------------G--------
1      -----------C---C---AT-NNNNNNNNNNN-----C-------TG----G---N-C-C-----C--A------------G--T-----
1      ----------A----N---NN-NNNNNNNNNNN-----------------------N-NNC-----C---------------G--------
1      ----A------C---C---AT------A----C-----------------------N---C----TC--A------------G--------
1      -----------C---C---AT------A----C---------------------------C----TC--A------------G--------
1      -----------C---C---AT----A-A----C-------------------------NNC-----C--A------------G--------
1      -----------C---C---AT------A----CG-------------------------NC-----C--N------------G--------
1      -----------C---C---AT------A----C----------------------C----C-----C--A------------G--------
1      -----------C---N---NN------A----C-----------------------N-NNC-----C--N------------G--------
1      -----------C---N---AT------A----C-----------------------N-NNC-----C--N------------G--------
2      -----------C---C---AT------A----C---------------------------C-----C--A------------G--------
1      ----------A----C-----------T----C-----------------------C--AC-----C---------------G--------
2      ----------A----C-----------T----C------A----------------C--AC-----C---------------G--------
1      ----------A----N-----------T----C--------T--------------N--AC-----C---------------G--------
```

```
1      ----------A----C-----------T----C-----------------------N--AC-----C---------------G--------
1      ----------A----------------T----C---------------------C-C--AC-----C---------A-----G--------
1      T----G---G----C-AGNA-AAA--CTACAA------C-------T----TGN----C-NNC-N----ACCCTCN-N-----CC----A-
1      T----G---G----C-AGNA-AAA--CTANNNN-----C-------T----TGN----C-NNC-N----ACCCTCN-N-----CC----AA
1      T----G---G---CC-AG-A-AAA--CTACAA------C-------TG---TGN----C---C------ACCCTC--N-----C----AA-
1      T----G----------AGNA-ANNNNNNNNNNN-----C-------T----TGN----C-NNC-N----ACCCTCN-N-----C-----A-
1      T------C-G------AGNA-AAA--CTACAA--A-T-C---G---T--T-TGN----C-NNC-N----ACCCTCN-N-----C-----A-
1      T--G---C-G------AGNA-AAA--CTACAA--A-T-C---G---T-GT-TGN----C-NNC-N----ACCCTCN-N-G---C-----A-
1      T----G---G---CC-AGNA-AAA--CTACAA------C-------T----TGN----C-NNC-N----ACCCTCN-N-----C-----A-
1      T----G---G---CC-AG-A-ANNNNNNNNNNN-----C-------T----TGN----C-NNC-N----ACCCTC--N-----C-----A-
2      T----G---G---CC-AGNA-ANNNNNNNNNNN-----C-------T----TGN----C-NNC-N----ACCCTCN-N-----C-----A-
1      T----G---G----C-AGNA-ANNNNNNNNNNN-----C-------T----TGN----C-NNC-N----ACCCTCN-N-----C---T-A-
1      T----G---G----C-AG-A-ANNNNNNNNNNN-----C-------T----TGN----C-CNC------ACCCTC--N-----C-----A-
9      T----G---G----C-AGNA-ANNNNNNNNNNN-----C-------T----TGN----C-NNC-N----ACCCTCN-N-----C-----A-
1      T----G---G----C-NGCA-NAA--CTACAA------C-------T----TGN----C-CGC-G----ACCCTCC-G-----C-T---A-
1      T----G---G----C-AG-A-AAA--CTACAA------C-------T----TG-----C-C-C------ACCCTC-------GC-----A-
1      T----G---G----C-AG-A-AAA--CTACAA------C-------T----TG-----C-C-C------ACCCTC--------C-----A-
1      T----G---G----C-AG-A-AAA--CTACAA------C-------T----TG-----C-N-C------ACCCTC--------C-----A-
1      T----G---G----C-AGNN-AAA--CTACAA------C-------T----TGN----N-CNC-N----ACCCTCN-N-----C-----A-
1      T----G---G----C-AG-A-AAA--CTACAA------C-------T----TGG----C-CNC------ACCCTCC-------C-----A-
2      T----G---G----C-AG-A-AAA--CTACAA------C-------T----TGG----C-C-C------ACCCTCC-------C-----A-
1      T----G---G----C-ANCA-NAA--CTACAA------C-------T----TGG----C-CNC-G----ACCCTCC-G-----C-----A-
1      T----G---G----C-NNCA-AAA--CTACAA------C-------T----TGG----C-CNC-N----ACCCTCC-G-----C-----A-
1      T----G---G----C-AGCA-AAA--CTACAA------C-------T----TGG----C-CGC-G----ACCCTCC-G-----C-----A-
1      T----G---G----C-AGCA-AAAG-CTACAA------C-------T----TGG----C-CGCTG----ACCCTCC-G-----C-----A-
```

`Sum=76, EP_ref is the reference genome (not included in the analysis, but included here for display)`

“N” represents bases that were not covered in the sequencing thus are missing. The most left column is the number of sequences with the displayed haplotype in that row.
