# Supplementary material for: Whole Genome Sequencing and Evolutionary Analysis of Human Papillomavirus Type 16 in Central China
Source: PLoS One. 2012 May 4;7(5):e36577. doi: 10.1371/journal.pone.0036577 (PMC3344914; doi:10.1371/journal.pone.0036577)
Supplement: Table S1 — HPV16 positive cervical patient information. (PDF) [file pone.0036577.s003.pdf]

Table S1. HPV16 positive cervical patient information

**Parameter**	**Value**	**HPV16 variants**	
		**As**	**E**
Age group	30-40	13	14
	41-50	8	12
	51-60	7	10
	>60	5	7
Pathology	Squamous cell carcinoma	31	38
	Adenocarcinoma	2	0
	Adenosquamous carcinoma	0	5
Clinical stage	Ia	3	1
	Ib/Ic	25	31
	II	4	4
Pathological grade	I	1	0
	II	9	12
	III	20	22
