# Supplementary material for: Whole Genome Sequencing and Evolutionary Analysis of Human Papillomavirus Type 16 in Central China
Source: PLoS One. 2012 May 4;7(5):e36577. doi: 10.1371/journal.pone.0036577 (PMC3344914; doi:10.1371/journal.pone.0036577)
Supplement: Table S2 — PCR primer for testing HPV presence. (PDF) [file pone.0036577.s004.pdf]

Table S2. PCR primer for testing HPV presence

Primer	Primer sequence 5’-3’	PCR Products (bp)
SPF1：		
SPF1A	GCICAGGGICACAATAATGG	186
SPF1B	GCIC.AGGGICATAACAATGG	
SPF1C	GCICAGGGICATAATAATGG	
SPF1D	GCICAAGGICATAATAATGG	
GP6+	GAAAAATAAACTGTAAATCATATTC	

I represents inosine
