# Supplementary material for: Whole Genome Sequencing and Evolutionary Analysis of Human Papillomavirus Type 16 in Central China
Source: PLoS One. 2012 May 4;7(5):e36577. doi: 10.1371/journal.pone.0036577 (PMC3344914; doi:10.1371/journal.pone.0036577)
Supplement: Table S3 — PCR primers for testing HPV types. (PDF) [file pone.0036577.s005.pdf]

Table S3. PCR primers for testing HPV types

HPV type	Primer direction	Primer sequence5’-3’	Gene	PCR products (bp)
-6	F	CTGTTTCGAGGCGGCTATC	E6	323
	R	TGGAGGTTGCAGGTCTAAT		
-11	F	GCGTGTGCCTGTTGCTTAGA	E6E7	218
	R	CCCTTCCACTGGTTATTTAG		
-16	F	ATGACTTTGCTTTTCGGGATTTAT	E6E7	335
	R	GCATGATTACAGCTGGGTTTCTC		
-18	F	AACCGAGCACGACAGGAACG	E7	368
	R	GGATGCACACCACGGACACA		
-26	F	TGACCTACGCTGCTACGAACAA	E7	294
	R	CCCGCCCCTCCTCATTT		
-31	F	ACCGTTGTGTCCAGAAGAAA	E6E7	
	R	CTAGTAGAACAGTTGGGGCACAC		419
-45	F	ACGACCCTACAAGCTACCAGATTT	E6	454
	R	TTGCTATACTTGTGTTTCCCTACG		
-56	F	TGGGGTGCTGGAGACAAACA	E7	271
	R	CTGCACCACAAACTTACACTCACA		
-57	F	ATACCCGAAATTGTTGACCT	E7	182
	R	TGCTCCAGATGCCTTATGT		
-58	F	CCAGGACGCAGAGGAGAAACC	E6	387
	R	CGACCCGAAATATTATGAAACCTT		
-94	F	GTAACGAACGGCGACGACTG	E6E7	205
	R	CCTATACGCTTGTTGTGCTGGTTC		
