# Supplementary material for: Whole Genome Sequencing and Evolutionary Analysis of Human Papillomavirus Type 16 in Central China
Source: PLoS One. 2012 May 4;7(5):e36577. doi: 10.1371/journal.pone.0036577 (PMC3344914; doi:10.1371/journal.pone.0036577)
Supplement: Table S4 — The PCR primers for the five samples that were checked with clone sequencing. (PDF) [file pone.0036577.s006.pdf]

Table S4. The PCR primers for the five samples that were checked with clone sequencing

HPV16 DNA fragment	Primers (F/R)
14-872	F: ATGTATAAAACTAAGGGCGTAACC
	R: TCAGCCATGGTAGATTAT
500-1780	F:ACCGGTCGATGTCTTG
	R: ATCATACACATTGGAGACACA
1734-2447	F: GAAAAATTGCTGTCTAAACTAT
	R: ACAGGGCACTGTAGCATCATC
2400-3724	F: TTAGCAGATGCCAAAATAGGT
	R: GTCCTGTCCAATGCCATGTAGA
3373-3893	F: TGAAATTATTAGGCAGCACTT
	R: AAAAGCACGCCAGTAATG
3862-4864	F: ATACTGCATCCACAACATTACTG
	R: TACTGTGTTAGGGTTTGTGCT
4764-5565	F: CTGGAGGGCATTTTACACTTT
	R: CCTGCATCAGCAATAATTGTA
5520-6356	F: TATAGTTCCAGGGTCTCC
	R: TTCTGACACCATTTTAATATA
6003-7154	F: TTAAATAAATTGGATGACACAGAA
	R: TTACAGCTTACGTTTTTTGCG
7028-7080	F: AGGACGCAAATTTTTACTACAAG
	R: ATGTCTGCTTTTATACTAACCG
