# Supplementary material for: Whole Genome Sequencing and Evolutionary Analysis of Human Papillomavirus Type 16 in Central China
Source: PLoS One. 2012 May 4;7(5):e36577. doi: 10.1371/journal.pone.0036577 (PMC3344914; doi:10.1371/journal.pone.0036577)
Supplement: Table S5 — The PCR primers for sequencing the HPV16 genomes. (PDF) [file pone.0036577.s007.pdf]

Table S5. The PCR primers for sequencing the HPV16 genomes

Gene	Genomic Region	Primers	Sequence(5'-3')	Product(bp)
E6	21-565	E6-F	AAACTAAGGGCGTAACCGAA	545
		E6-R	GCATGATTACAGCTGGGTTTCTC	
E7	500-872	F500	ACCGGTCGATGTATGTCTTG	372
		R872	TCAGCCATGGTAGATTAT	
E1	2014-2360	F2014	GACACTAATAGTAATGCAAGT	346
		R2360	TATTACAGACCCTTGCAGAA	
	2203-2447	F2203	AGGTATCAAGGTGTAGAG	244
		R2447	ACAGGGCACTGTAGCATCATC	
	1333-1780	F1333	TGTAGTCAGTATAGTGGTGGAA	447
		R1780	ATCATACACATTGGAGACACA	
	1734-2184	F1734	GAAAAATTGCTGTCTAAACTAT	450
		R2184	GCTTCCAATCACCTCCATCATCTA	
		R2184	GCTTCCAATCACCTCCATCATCTA	
E2	3355-3570	F3355	TAGCAGCAACGAAGTATCCT	215
		R3570	GTGTGAGCTGTTAAATGCAG	
	2837-3020	F2837	ACCATATAGACTATTGGAA	183
		R3020	TTACTATATTGTGAGTTAT	
	2951-3320	F2951	AAGAATAAAGCATTACAAGC	369
		R3320	GCATGAACTTCCCATACT	
	3701-3893	F3701	CGTCTACATGGCATTGGAC	192
		R3893	AAAAGCACGCCAGTAATG	
E5	3862-4130	F3862	ATACTGCATCCACAACATTACTG	268
		R4130	GTAACAATTACATTATGTA	
	3885-4436	F3885	GCGTGCTTTTTGCTTTGCTTTGT	551
		R4436	CGCCTGTACCCGACCCTGTT	
	3896-4436	F3896	AACATTACTGGCGTGCTTTTTG	540
		R4436	CGCCTGTACCCGACCCTGTT	
L2	4522-4864	F4522	TCCTGTGGGCCCTTCTGAT	342
		R4864	TACTGTGTTAGGGTTTGTGCT	
	5292-5570	F5292	CACATGCAGCCTCACCTACT	278
		R5570	GTCACCTGCATCAGCAATA	
	4959-5351	F4959	TTGTAACCACTCCCACTAAAC	392
		R5351	AGTCATCTGCATAAATATCATA	
	4136-4479	F4136	TGTTGTATACCATAACTTAC	343
		R4479	GCTGTGGGAGGCCTTGTTC	
	4785-5131	F4785	TTCATCATCCACTATTAGTACA	346
		R5131	TACGCCTAGAGGTTAATGCT	
L1	6187-6486	F6187	TGATTGTCCACCATTAGA	299
		R6486	GTAGACCCAGAGCCTTTA	
	6351-6602	F6351	TGTCAGAACCATATGGCGAC	251
		R6602	CATTATTGTGGCCCTGTGCT	

	7028-7375	F7028	AGGACGCAAATTTTTACTACAAG	347
		R7375	TATACAATGAATAACCACA	
	6582-7033	MY11	ACCGGTCGATGTATGTCTTG	451
		MY09	TCAGCCATGGTAGATTAT	
NCR	4010-4307	F4010	CCTCTGCGTTTAGGTGTTTTA	297
		R4307	CTGCCTGTTTGCATGTTTTAT	
LCR	7142-7459	F7142	ACGTAAGCTGTAAGTATTG	317
		R7459	CGAATTCGGTTGAAGCTACA	
	7460-7863	LCR-F	TCGGTTGCATGCTTTTTGGC	403
		LCR-R	CGGTTTGCACACACCCATGT	
	7781-100	F7781	GTTAGTCATACATTGTTCAT	225
		R100	AGTTCTCTTTTGGTGCATA	
