# Supplementary material for: Whole Genome Sequencing and Evolutionary Analysis of Human Papillomavirus Type 16 in Central China
Source: PLoS One. 2012 May 4;7(5):e36577. doi: 10.1371/journal.pone.0036577 (PMC3344914; doi:10.1371/journal.pone.0036577)
Supplement: Table S6 — PCR reaction conditions. (PDF) [file pone.0036577.s008.pdf]

## Table S6.PCR reaction conditions

| PCR reaction conditions | | | | |
|---|---|---|---|---|
| Enzyme | System | | Procedure | Samples |
| KOD HotStart DNA Polymerase | 10×PCRbuffer | 5ul | 95℃ 2min | clone sequencing |
| | dNTP（2mM） | 5ul | 95℃ 20s | |
| | Primer forward(10uM ) | 1.5ul | Tm 10s | |
| | Primer reverse(10uM) | 1.5ul | 70℃ 20s | |
| | Enzyme | 1ul | 70℃ 1min | |
| | $MgSO_4$（25mM） | 3ul | 4℃ forever | |
| | Template DNA | 100-200ng | | |
| | $ddH_2O$ | | | |
| | total | 50ul | 38cycles | |
| Platinum® PCR SuperMix | SuperMix | 40ul | 94℃ 2min | clone sequencing |
| | Primer forward(10uM ) | 1.5ul | 94℃ 15s | |
| | Primer reverse(10uM) | 1.5ul | Tm 30s | |
| | Template DNA | 100-200ng | 68℃ 1min | |
| | | | 68℃ 5min | |
| | | | 4℃ forever | |
| | total | 45ul | 38cycles | |
| TaKaRa rTaq DNA Polymerase | 10×PCRbuffer | 4ul | 95℃ 3min | Whole genome sequencing |
| | dNTP（10mM） | 0.5ul | 95℃ 40s | |
| | Primer forward(10uM ) | 0.8ul | Tm 40s | |
| | Primer reverse(10uM) | 0.8ul | 72℃ 40s | |
| | Enzyme | 0.4ul | 72℃ 1min | |
| | Template DNA | 100-200ng | 4℃ forever | |
| | $ddH_2O$ | | | |
| | total | 40ul | 38cycles | |
| HotStarTaq Master Mix Kit | 10×PCRbuffer | 4ul | 95℃ 15min | Whole genome sequencing ,E7 ,E6,L1(SPF/GP6+) amplification |
| | dNTP（10mM） | 0.6ul | 94℃ 40s | |
| | Primer forward(10uM ) | 1.6ul | Tm 40s | |
| | Primer reverse(10uM) | 1.6ul | 72℃ 30s | |
| | Enzyme | 0.2ul | 72℃ 1min | |
| | $MgCL_2$（25mM） | 0.8ul | 4℃ forever | |
| | Template DNA | 50ng | | |
| | $ddH_2O$ | | | |
| | total | 40ul | 38cycles | |
| Trans Taq HiFi DNA | 10×PCRbuffer | 4ul | 95℃ 3min | Whole genome sequencing |
| | dNTP（10mM） | 0.5ul | 95℃ 40s | |

| | | | | |
|---|---|---|---|---|
| Polymerase | Primer forward(10uM ) | 0.8ul | Tm 40s | |
| | Primer reverse(10uM) | 0.8ul | 72℃ 40s | |
| | Enzyme | 0.3ul | 72℃ 1min | |
| | Template DNA | 100-200ng | 4℃ forever | |
| | $ddH_2O$ | | | |
| | total | 40ul | 38cycles | |
| 2XPCR TaqMix | 2XPCR TaqMix | 10ul | 94℃ 2min | PCR during Clone sequencing |
| | Primer forward(10uM ) | 1ul | 94℃ 30s | |
| | Primer reverse(10uM) | 1ul | Tm 30s | |
| | Template DNA | 100-200ng | 72℃ 75s | |
| | $ddH_2O$ | | 72℃ 5min | |
| | | | 4℃ forever | |
| | total | 20ul | 38cycles | |
