# Supplementary material for: Whole Genome Sequencing and Evolutionary Analysis of Human Papillomavirus Type 16 in Central China
Source: PLoS One. 2012 May 4;7(5):e36577. doi: 10.1371/journal.pone.0036577 (PMC3344914; doi:10.1371/journal.pone.0036577)
Supplement: Text S1 — Supporting methods. (DOC) [file pone.0036577.s009.doc]

**Text S1**

**1) Sample collection**

The cancer samples we collected in this study were all from patients with cervical cancers where the whole uterus was removed from the patients during the cancer therapy. Before the surgery, the patients haven’t received any chemotherapy. The tumor samples in this study were a small proportion of the patient samples from this hospital where surgery (i.e. removing uterus) was chosen as an effective treatment. Later stage cancer patients will directly go to radiation therapy without surgery. Most of the cancer types are squamous cell carcinoma even though a few other types are also present (see Table S1).

**2) Sample processing**

After obtaining the tissue samples from the local hospital, continuous slices of 5um each were cut from each sample. The slides were subsequently collected into EP tubes. To avoid cross contamination between samples, the microtome blade was carefully cleaned. Blank paraffin samples were tested to ensure the cleanness of the blades.

Subsequent sample processing follows strict laboratory rules to ensure good quality in the experimental procedures and avoid cross contaminations. For example, DNA extraction, PCR reaction and electrophoresis were done in separate rooms. Laboratory personnel were instructed to wear gloves when handling the samples and the experimental area were regularly cleaned before beginning work. In addition, a regular procedure of inspecting the experiment area surfaces (cotton bud was first applied to various of surfaces, e.g. lab benches, subsequently they were soaked into deionized water overnight. HPV detection was applied to the supernatant. Experiments were allowed only when negative results were observed). Additionally, we also used a mouse liver tissue as an internal control together with the cancer samples. Experimental procedure was preceded only negative results are observed from these internal controls (data not shown).

**3) HPV detection and classification**

PCR primers were designed to detect the presence of HPV L1 region (Primer set is presented in the Table S2). After finding the samples with the presence of HPVs, two parallel strategies were used to identify the HPV types in the cancer tissues at two different labs. The first strategy is based on the sequenom platform at Beijing Genome Institute at Shenzhen where amplicons based on the L1 region were identified using mass spectrum technology. The second strategy is the PCR amplification targeting on specific E6/E7 regions and was conducted in the lab at Beijing (Table S3). The commercial service from BGI-Shenzhen detects much wider range of HPV types than our lab based experiments (Table S3), however, the results from the two labs were matching with each other very well on HPV16 subtype (data not shown).

Of the 94 cancer samples, we were able to detect HPV in 87 samples. Of these 87 specimens, 80 were positive for HPV16. In addition to HPV type 16, we were also able to find HPV18(11.7%), 31(1%), 35(1%), 39(1%), 45(1%), 52(1%), 58(13.8%), 59(3.2%) and 66(3.2%). Infection with only one single type was found in 62 samples and the other 22 specimens showed multiple infection.

**4) PCR, Sanger sequencing and quality control**

Of these 80 cases that tested HPV16 positive, four cases had too low dosage of HPV16 to allow efficient PCR reactions and thus were dropped from the current study. All the other 76 sequences we obtained for this study are from PCR/Sanger sequencing (Table S5). In order to check the reliability of our data, we selected five samples for clone sequencing across the genome (Table S4). For these five samples, we repeated all the experimental step by starting from sample slicing and DNA extraction. In the clone sequencing stage, high fidelity Platinum® PCR SuperMix High Fidelity and KOD HotStart DNA Polymerase kit were used to ensure minimum possible amplification errors. The PCR products were subsequently cloned into vectors and 3-5 clones were chosen for Sanger sequencing. All the clones matched with each other and the results from direct PCR and Sanger sequencing (data not shown).

**5) PCR sequencing from Formalin-fixed cancer specimens**

Even though Formalin-fixed tissues often have potential problems of DNA fragmentation

and DNA-protein cross-linking by formaldehyde exposure, we were able to detect HPV in 87 out of 94 samples. There are several factors that contributed to the high success rate in extracting the viral genome in this study.

a) First of all, the samples we collected, even though formalin-fixed, are relatively young in age(less than 3 years). Electrophoresis analysis indicated that the DNA fragment lengths were quite good ( ~ 1000bp).

b) We used DNA extraction kit specifically designed for formalin-fixed tissues.

c) We designed PCR primers that will amplify short DNA segments from the specimens and high fidelity taq enzyme were used to ensure integrity of the amplified DNA sequences.

d) The PCR segments overlap with each other and a large proportion of the genome is sequenced multiple times.

e) PCR products were sequenced from both ends of the segments and checked against each other.

Of all the PCR regions, there are three segments that failed in the PCR amplification. These were two segments within E1 region of size 475 bp and 309 bp and they failed in most of the clinical samples. The other segment of size 928 bp in the E2 gene cannot be amplified only in a subset of 31 samples (Figure 2).

Even though small parts of the E1 and E2 genes failed in the PCR/Sequencing, most of the experimental procedures show good results. Sequences from both ends and overlapping regions show very good consistency with each other (data not shown). This indicates that HPV16 is the predominant type in the clinical specimen and cross infection is not substantial.
